# Breast sensibility in bilateral autologous breast reconstruction with unilateral sensory nerve coaptation

**DOI:** 10.1007/s10549-020-05645-y

**Published:** 2020-04-28

**Authors:** Ennie Bijkerk, Sander M. J. van Kuijk, Arno Lataster, René R. W. J. van der Hulst, Stefania M. H. Tuinder

**Affiliations:** 1 Department of Plastic and Reconstructive Surgery, Maastricht University Medical Center, P.O. Box 5800, 6202 AZ Maastricht, the Netherlands; 2 GROW–School for Oncology and Developmental Biology, Maastricht University, Maastricht, The Netherlands; 3 Department of Clinical Epidemiology and Medical Technology Assessment (KEMTA), Maastricht University Medical Center, Maastricht, The Netherlands; 4 Department of Anatomy and Embryology, Maastricht University, Maastricht, The Netherlands

**Keywords:** Sensory nerve coaptation, Autologous breast reconstruction, Breast cancer, Sensation, Perforator flap

## Abstract

**Background:**

Patient satisfaction after breast reconstruction is dependent on both esthetics and functional outcomes. In an attempt to improve breast sensibility, a sensory nerve coaptation can be performed. The aim of this study was to objectify the sensory recovery in patients who, by chance, underwent bilateral autologous breast reconstruction with one innervated and one non-innervated flap. It must be emphasized that the intention was to coaptate the sensory nerves on both sides.

**Methods:**

The cohort study was carried out in the Maastricht University Medical Center between August 2016 and August 2018. Patients were eligible if they underwent bilateral non-complex, autologous breast reconstruction with unilateral sensory nerve coaptation and underwent sensory measurements using Semmes–Weinstein monofilaments at 12 months of follow-up. Sensory outcomes were compared using *t* tests.

**Results:**

A total of 15 patients were included, all contributing one innervated and one non-innervated flap. All patients had a follow-up of at least 12 months, but were measured at different follow-up points with a mean follow-up of 19 months. Sensory nerve coaptation was significantly associated with better sensation in the innervated breasts and showed better sensory recovery over time, compared to non-innervated breasts. Moreover, the protective sensation of the skin can be restored by sensory nerve coaptation.

**Conclusions:**

The study demonstrated that sensory nerve coaptation leads to better sensation in the autologous reconstructed breast in patients who underwent bilateral breast reconstruction and, by chance, received unilateral sensory nerve coaptation.

## Introduction

Due to improved diagnostic tools and advancements in the therapeutic options, the survival rate of breast cancer continues to rise [1]. One of the major cornerstones in breast cancer treatment remains a mastectomy, which is performed in approximately 30–40% of all breast cancer patients [2]. In addition, the prevalence of gene mutation carriers, such as BRCA-1 and BRCA-2, is rising as well. These gene mutation carriers are often young women who choose to undergo risk-reducing mastectomies [3]. *More* women undergo breast cancer surgery at a *young* age and live *longer* afterwards. As a consequence, disabilities associated with a mastectomy can greatly affect their quality of life (QoL).

Approximately, a quarter of all patients opt for immediate breast reconstruction following mastectomy [2]. For a long time, the main focus of breast reconstructive surgery was the esthetic outcome. Excellent cosmetic results can be achieved and many studies have shown the positive impact of breast reconstruction on the QoL of breast cancer survivors [4]. However, QoL is determined not only by esthetic outcome, but also by functional aspects such as sensation. A pilot study by Cornelissen et al. showed a positive association between sensation in the reconstructed breast and QoL [5]. To restore sensation in the reconstructed breast, a sensory nerve coaptation can be performed during autologous breast reconstruction. Promising results have been demonstrated with better sensation in women who received innervated deep inferior epigastric perforator (DIEP) flaps, compared to women who received non-innervated DIEP flaps [6]. The primary aim of this study was to evaluate sensory recovery in the reconstructed breasts of patients who underwent bilateral autologous breast reconstruction with only a unilateral sensory nerve coaptation. In addition, success rates of intended sensory nerve coaptations were assessed.

## Materials and methods

A partially retrospective and partially prospective cohort study was carried out in the Maastricht University Medical Center, the Netherlands, between August 2016 and August 2018. The study was conducted in compliance with the world medical association Declaration of Helsinki (2013) [7] and reported in accordance with the STROBE Statement [8]. Ethical approval was obtained from the local Medical Ethical Committee (METC) of Maastricht University. Written informed consent was obtained from each patient.

### Study population

All patients who underwent autologous breast reconstruction in the Maastricht University Medical Center between August 2016 and August 2018 were retrospectively screened for inclusion criteria. Inclusion criteria were as follows: a bilateral breast reconstruction with a unilateral sensory nerve coaptation and a follow-up period of 12 months or more. It must be emphasized that the intention was always to bilaterally coaptate a sensory nerve, and it was never intentionally coaptated unilaterally, due to ethical aspects. Exclusion criteria were as follows: absent postoperative sensory measurements, postoperative radiotherapy on the flap, complex breast reconstruction techniques (such as a stacked four-flap reconstruction), neurological conditions that could affect sensation (such as diabetes mellitus), and active smoking. Peripheral neuropathy due to chemotherapy was not considered to be an exclusion criterion. In addition, patients who underwent bilateral breast reconstruction with bilateral sensory nerve coaptation were retrospectively screened to see if they underwent unilateral revision surgery. In case of revision surgery, flap survival was the first priority and the sensory nerve coaptation was sacrificed, resulting in a bilateral breast reconstruction with a unilateral sensory nerve coaptation. Demographic and medical data were collected for each patient included.

### Surgical technique

Sensory nerve coaptations were performed according to the technique introduced by Spiegel et al. [9, 10] The recipient nerve is the anterior cutaneous branch (ACB) of the second or third intercostal nerve (ICN), as these branches are localized in the same surgical field as the recipient vessels. The donor nerve was a sensory branch of the 10th to 12th intercostal nerve in DIEP flaps [11, 12] and a branch of the lateral femoral cutaneous nerve (LFCN), and sometimes an anterior cutaneous branch of the femoral nerve (ACFN) in LTP flaps [13, 14]. The main criterion for selecting the donor nerve was a localization in the vicinity of the dominant perforator, so that a tensionless coaptation could be performed. In the abdomen, the dominant perforator is generally located in the medial row, within 3 cm of the umbilicus [15]. In the lateral thigh, the LFCN is most often located cranially to the perforator and enters the flap anteriorly [16]. The LTP flaps could therefore be transposed to the thorax without rotation for flap inset: the cranial border of the flap formed the upper pole and the anterior border of the flap formed the medial side of the breast. In a bilateral DIEP flap breast reconstruction, the ipsilateral flaps were rotated 90°, so that the medial border of the flaps formed the inframammary fold and the lateral borders of the flaps filled the upper pole of the breast. Another criterion is an optimal match in the diameter of the donor and recipient nerves. A direct end-to-end nerve coaptation without tension was performed with two 9-0 nylon epineural microsutures and fibrin sealant. No nerve conduits or grafts were used.

### Sensory measurements

In each patient, sensory measurements were prospectively collected to assess the cutaneous pressure sensitivity threshold in the breasts, using a Semmes–Weinstein monofilament 20-piece full kit. The index values of the monofilaments ranged from 1.65 (thinnest monofilament) to 6.65 (thickest monofilament). Each index value represents the logarithm of the force in milligrams required to bend the monofilament into a C-shape. A thinner monofilament requires less pressure to bend and, therefore, corresponds to a lower pressure sensitivity threshold of the skin and, thus, better sensation.

**Fig. 1:**
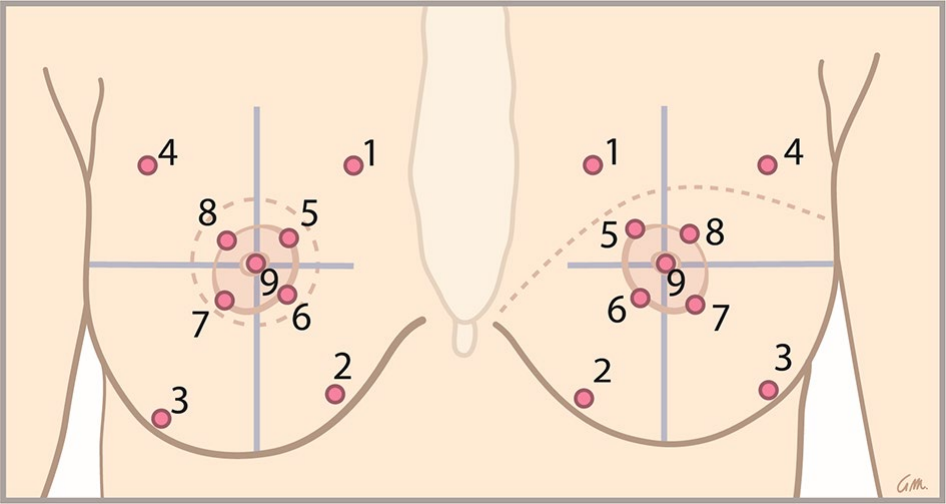
The nine areas of either immediate (on the left) or delayed reconstructed (on the right) breasts that were tested using Semmes–Weinstein monofilaments. The areas were determined using anatomical landmarks

The patients were in supine position, and had their eyes closed. Nine different measurement points (Fig. 1) were randomly tested to assess breast sensibility. Each point was measured three times in a row, 1.5 s each time. Perpendicular pressure was applied until the monofilament was C-shaped. Testing started with the thinnest monofilament and proceeded with thicker monofilaments until the patient identified touch.

Using Semmes–Weinstein monofilaments, sensation can be divided into 1 of 5 levels, each marked a color on the rods. Green represents normal touch (index values ranging from 1.65 to 2.83), blue represents diminished light touch (index values ranging from 3.22 to 3.61), purple represents diminished protective sensation (index values ranging from 3.84 to 4.31), and red represents loss of protective sensation (index values ranging from 4.56 to 6.45) and deep pressure sensation only (index value 6.65) [17]. These levels were maintained in the current study. A cut-off-value of 4.56 was maintained as clinically relevant, as this value and above corresponds to a loss of protective function.

### Success rates of sensory nerve coaptations

Whenever sensory nerve coaptation was performed in a bilateral breast reconstruction procedure, the intention was always to perform it bilateral. The patients included in the study received bilateral breast reconstruction, but did not receive bilateral sensory nerve coaptation because surgical aspects, such as flap orientation, were a limiting factor. To evaluate the role of a possible learning curve for successful bilateral sensory nerve coaptation, the overall success rate and the individual success rates for bilateral sensory nerve coaptation of the participating surgeons were calculated. The total number of breasts that were initially intended to be innervated in bilateral breast reconstruction during the study period was compared to the total number of reconstructed breasts that were effectively innervated. In addition, subgroup analyses were performed to evaluate success rates of nerve coaptations between reconstructive surgeons.

### Statistical analyses

A sample size could not be calculated, due to the lack of data that were available on the sensory recovery in innervated and non-innervated reconstructed breasts within the same patient population. Therefore, all patients that fit the eligibility criteria were included within the study period.

Continuous variables were presented as mean and standard deviation (SD) or median with interquartile range (IQR), depending on the distribution of the data. Continuous variables were compared using a paired *t* test. Nominal variables were presented as absolute numbers and percentages and compared with a McNemar’s test. In case of missing values, complete case analyses were performed.

Each patient contributed two breasts to the database, one *with* and one *without* sensory nerve coaptation. All patients were measured repeatedly during the follow-up period. Therefore, longitudinal analysis was required, as length of follow-up and sensory nerve coaptation were considered within-subject variables. Generalized estimating equations (GEE) were used to estimate the differences in monofilament values between innervated and no-innervated reconstructed breasts. The crude estimated differences were adjusted for clinically relevant confounders, such as the length of follow-up, timing of reconstruction, and the number of previously undergone breast surgeries. Because all women participated in both investigated groups, characteristics are subdivided into those that are relevant on *patient level* and those that are relevant on *breast level*. However, because within patients some of these characteristics on *breast level* did differ, it was adjusted for in the GEE model. GEE analyses were also used to estimate the association between the length of follow-up and sensory recovery in the reconstructed breasts. Moreover, grouped scatterplots were made and LOESS curves were fit to illustrate the sensory recovery over time in both innervated and non-innervated breasts.

The primary outcome was the difference in sensation between the innervated and non-innervated reconstructed breasts. The monofilament index values were assessed separately for each measurement point, native and flap skin and for the whole breast, and analyzed accordingly. In general, the native skin is represented by measurement points 1 to 4 in immediate breast reconstructions, and by measurement points 1 and 4 in delayed breast reconstructions. The flap skin is in immediate breast reconstructions represented by measurement points 5 to 9 and in delayed breast reconstructions by measurement points 2, 3, and 5 to 9 (Fig. 1). For all patients who diverged from standard mapping, the mean values of native and flap skin were manually calculated (e.g., if the skin islands were removed during correction surgery). Preoperatively, measurement points 1 to 4 represent the peripheral skin. Measurement points 5 to 9 represent the nipple–areola complex (NAC).

A *p* value of < 0.05 was considered statistically significant. All analyses were performed using IBM® SPSS® Statistics for Windows (Version 25.0, released 2017. Armonk, NY: IBM Corp).

## Results

### Characteristics

**Fig. 2:**
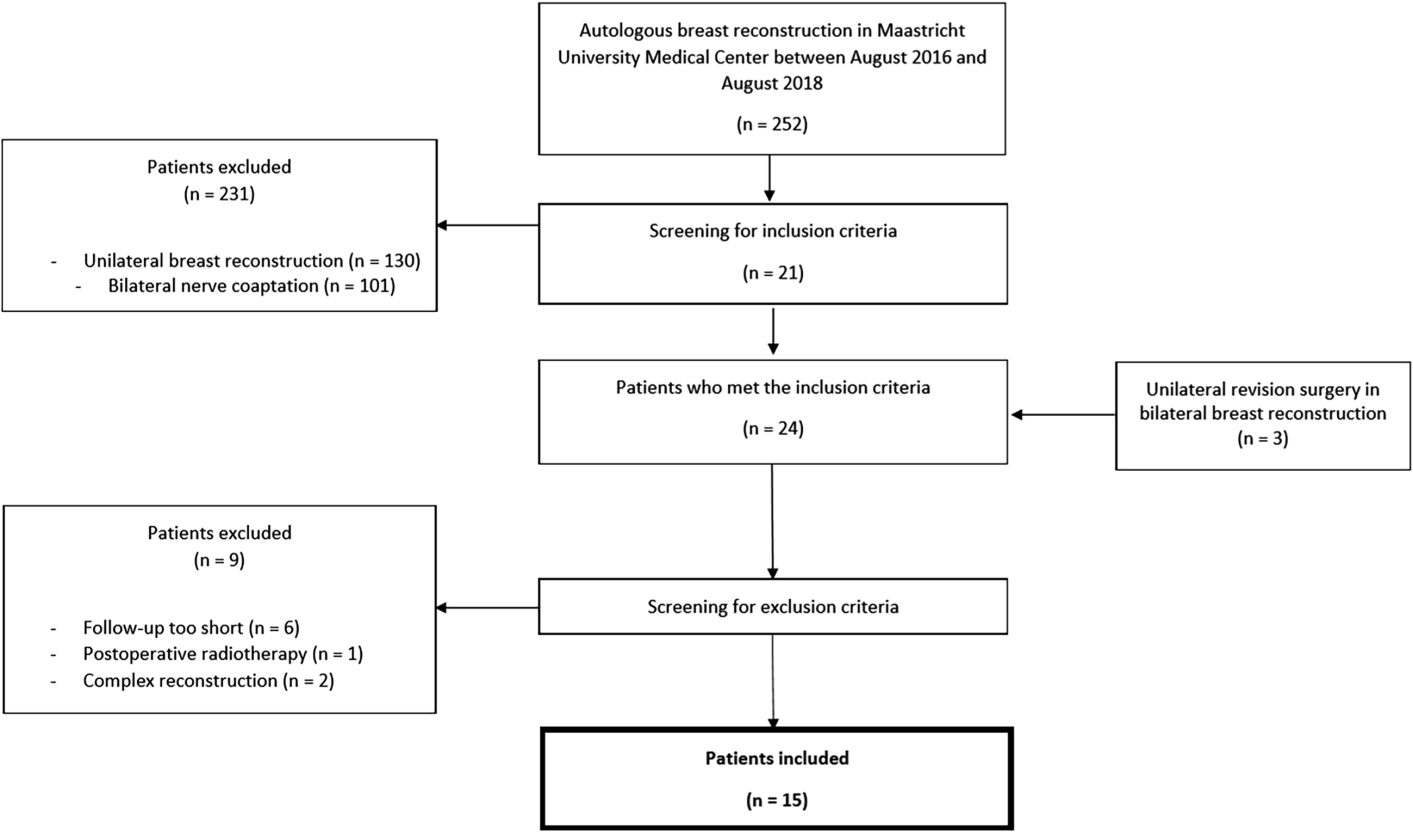
Flowchart of patient inclusion

A total of 252 patients underwent autologous breast reconstruction in the Maastricht University Medical Center between August 2016 and August 2018. Of those patients, 122 patients underwent a bilateral breast reconstruction, of which 21 patients received unilateral sensory nerve coaptation. Three patients were added to the study cohort because they initially received bilateral breast reconstruction with bilateral sensory nerve coaptation, but underwent unilateral revision surgery afterwards. Eighteen of these 24 patients had a follow-up of at least 12 months. One patient was excluded because of postoperative radiotherapy on the flaps. Two patients were excluded because they received a complex reconstructive technique: one patient underwent a stacked four-flap breast reconstruction, and one patient previously underwent combined implant-based and autologous breast reconstruction, using a latissimus dorsi (LD) flap. In total, 15 patients were included in the current study (Fig. 2). Twelve patients underwent DIEP flap breast reconstruction, three patients underwent lateral thigh perforator (LTP) flap breast reconstruction. The remaining patient characteristics are summarized in Table 1. The history of chemotherapy and radiotherapy was unknown for 2 and 3 patients, respectively. Information of the ischemia time and the flap weight was missing for 2 patients and 1 patient, respectively. No differences were found between the innervated and non-innervated flaps regarding the characteristics on *breast level*.

**Table 1:** Characteristics of the study population

*Patient level*	*N* = 15 (%)
Age in years (mean ± SD)	49 ± 13
BMI in kg/m^2^ (mean ± SD)	26.88 ± 3.33
Follow-up in months (mean, ± SD)	18.87 ± 5.18
History of chemotherapy	7 (46.7)
Flap type	
DIEP	12 (80)
LTP	3 (20)
Nipple reconstruction	8 (53.3)

Breast level	Innervated	Non-innervated	*p* value
	*N* = 15 (%)	*N* = 15 (%)	
History of radiotherapy	3 (20)	3 (20)	1.000
Number of previous breast surgeries (mean ± SD)	3.31 ± 0.85	3.00 ± 1.00	0.364
Timing			0.670
Immediate	7 (46.7)	8 (53.3)	
Delayed	8 (53.3)	7 (46.7)	
Ischemia time (mean ± SD)	45.9 ± 13.2	59.0 ± 63.1	0.508
Flap weight (mean ± SD)	555 ± 152	540 ± 148	0.147
Correction surgeries			
First correction surgery	13 (86.7)	10 (66.7)	0.082
Second correction surgery	4 (26.7)	2 (13.3)	0.164
Minor breast complications	3 (20)	3 (20)	0.310
Mastectomy skin necrosis	1 (6.7)	1 (6.7)	
Hematoma/ecchymosis	1 (6.7)	1 (6.7)	
Venous thrombosis	0	1 (6.7)	
Fat necrosis	1 (6.7)	0	

Variables are presented as absolute numbers and percentages or as mean and standard deviation (SD)

*BMI* body mass index, *DIEP* deep inferior epigastric perforator, *LTP* lateral thigh perforator

### Surgical details

The characteristics of the flaps are summarized in Table 2. In one flap, the superficial inferior epigastric artery (SIEA) was used, because of a dominant superficial system. In two flaps in the same patient, one perforator from the lateral row was used. In the remaining flaps, perforators from the medial row were used. In all cases, the flaps were harvested from the ipsilateral side for reconstruction of the breasts. In this way, vascular anastomosis and sensory nerve coaptation without tension were achieved.

**Table 2:** Flap characteristics

Perforator position*	*N* = 30 (%)
DIEP flaps	*N* = 24 (%)
Medial row	21 (87.5)
Lateral row	2 (8.3)
SIEA	1 (4.2)
LTP flaps	*N* = 6 (%)
Posterior septum	6 (100)
Anterior septum	0 (0)
No. of perforators	
1	24 (80.0)
2	4 (13.3)
3	2 (6.7)
Laterality of the donor site	
Ipsilateral	30 (100)
Contralateral	0 (0)

*DIEP* deep inferior epigastric perforator, *LTP* lateral thigh perforator, *SIEA* superficial inferior epigastric artery

*Percentages of perforator position are reported as a fraction of the total amount of DIEP and LTP flaps, respectively

### Sensory recovery in the reconstructed breasts

The preoperative measurements of each area, the peripheral skin, and the NAC are summarized in Table 3. Naturally, the breasts were postoperatively classified as either innervated or non-innervated. In retrospect, the preoperative values between the two groups did not differ significantly.

**Table 3:** Monofilament values per area, peripheral/native skin, Nipple-Areola-Complex/flap skin and total breast skin of preoperative measurements and at maximum follow-up

Area	Preoperative innervated	Postoperative innervated	*p* value	Preoperative non-innervated	Postoperative non-innervated	*p* value
1	2.36 (2.36–3.84)	2.44 (2.36–4.21)	0.146	2.59 (2.36–3.42)	3.34 (2.36–4.97)	0.059
2	2.40 (2.36–3.59)	3. 61 (2.42–4.79)	*0.004*	2.60 (2.36–3.84)	4.17 (3.51–4.82)	*0.004*
3	2.36 (2.36–3.69)	4.32 (2.83–5.18)	*0.001*	2.83 (2.36–4.02)	4.74 (3.84–5.18)	< *0.001*
4	2.36 (2.36–3.77)	3.70 (2.36–4.69)	0.065	2.60 (2.36–3.78)	4.24 (3.01–4.74)	*0.008*
Native	2.43 (2.36–3.66)	3.37 (3.02–3.64)	0.077	2.81 (2.36–3.67)	4.08 (3.64–4.93)	*0.003*
5	3.22 (2.44–4.57)	4.31 (3.78–5.25)	*0.011*	3.73 (2.36–4.17)	5.13 (4.93–5.57)	< *0.001*
6	3.22 (2.36–4.26)	4.24 (3.61–5.17)	*0.031*	3.73 (2.48–4.09)	5.13 (4.31–5.46)	*0.007*
7	3.03 (2.36–4.60)	4.53 (3.78–5.18)	*0.024*	3.73 (2.54–4.08)	5.00 (4.56–5.56)	*0.001*
8	3.53 (2.48–4.74)	4.65 (3.78–5.25)	0.062	3.84 (2.54–4.17)	5.06 (4.70–5.88)	< *0.001*
9	3.53 (2.48–4.74)	4.17 (3.69–5.15)	0.170	3.42 (2.82–4.28)	5.18 (4.78–5.57)	*0.001*
Flap	3.38 (2.62–4.43)	4.42 (3.67–5.13)	*0.025*	3.73 (2.52–4.10)	5.06 (4.60–5.44)	< *0.001*
Total	3.15 (2.54–4.10)	4.05 (3.52–4.76)	*0.010*	3.27 (2.45–3.86)	4.62 (4.35–5.08)	< *0.001*

Monofilament values are shown as median and interquartile range (IQR). Statistically significant p values are indicated in *italic*

The preoperative and postoperative monofilament values at maximum follow-up were compared for the innervated and non-innervated breasts (Table 3). Sensation was significantly impaired after non-innervated breast reconstruction in all areas, except area 1 (*p* < 0.008 and *p* = 0.059, respectively). In contrast, the innervated breasts showed significant impaired sensation in only 5 out of the measured 9 areas. This indicates that in almost half of the reconstructed breast (areas 1, 4, 8, and 9, *p* values 0.146, 0.065, 0.062, and 0.170, respectively) as well as the mean monofilament value of the native skin (*p* = 0.077), sensation was comparable to the preoperative values. Thus, sensation in the innervated breasts reached near normal levels, in both native skin (areas 1 and 4) and flap skin (areas 8 and 9).

The postoperative monofilament values were lower in the innervated breasts in all areas compared to the non-innervated breasts. Moreover, the postoperative monofilament values in the native skin were notably lower compared to those found in the flap skin in both innervated and non-innervated breasts (native: 3.37, IQR 3.02–3.64 in innervated breasts; 4.08 IQR 3.64–4.93 in non-innervated breasts, and flap: 4.42, IQR 3.67–5.13 in innervated breasts; 5.06, IQR 4.60–5.44 in non-innervated breasts).

### Sensory nerve coaptation and sensory recovery

The crude and adjusted regression coefficients of the association between sensory nerve coaptation and mean monofilament index values are reported in Table 4. Without adjusting for characteristics that were considered clinically relevant, sensory nerve coaptation was significantly associated with a lower mean monofilament values in the flap skin: areas 5, 6, and 8 (*p* values 0.022, 0.017, and 0.032, respectively) and the mean flap skin (mean flap skin difference − 0.3; *p* = 0.003).

**Table 4:** Crude and adjusted regression coefficients of the association between sensory nerve coaptation and mean monofilament scores per area

	Crude coefficients	95% CI	*p* value	Adjusted coefficients^†^	95% CI	*p* value
1	− 0.068	− 0.517 to 0.382	0.768	− 0.219	− 0.679 to 0.241	0.350
2	− 0.093	− 0.445 to 0.258	0.603	− 0.244	− 0.542 to 0.053	0.107
3	0.021	− 0.293 to 0.335	0.896	− 0.076	− 0.380 to 0.228	0.623
4	0.017	− 0.471 to 0.506	0.944	− 0.033	− 0.568 to 0.502	0.903
Native skin	− 0.057	− 0.428 to 0.313	0.761	− 0.118	− 0.486 to 0.250	0.531
5	− 0.365	− 0.678 to − 0.052	*0.022*	− 0.488	− 0.776 to − 0.200	*0.001*
6	− 0.357	− 0.651 to − 0.064	*0.017*	− 0.499	− 0.779 to − 0.220	< *0.001*
7	− 0.172	− 0.422 to 0.078	0.178	− 0.268	− 0.531 to − 0.005	*0.046*
8	− 0.314	− 0.601 to − 0.027	*0.032*	− 0.449	− 0.744 to − 0.155	*0.003*
9	− 0.282	− 0.580 to 0.016	0.064	− 0.374	− 0.655 to − 0.093	*0.009*
Flap skin	− 0.384	− 0.640 to − 0.128	*0.003*	− 0.439	− 0.734 to − 0.145	*0.003*
Total skin	− 0.185	− 0.404 to 0.034	0.98	− 0.287	− 0.458 to − 0.115	*0.001*

Statistically significant p values are indicated in *italic*

^†^Adjusted for length of follow up in months, timing of breast reconstruction (immediate versus delayed), type of flap (DIEP versus LTP) and history of radiotherapy

Adjustment for the length of follow-up in months, the timing of reconstruction (immediate versus delayed), and a history of radiotherapy showed that sensory nerve coaptation was significantly associated with lower monofilament values in all flap skin areas, the mean flap skin (mean flap skin difference − 0.439; *p* = 0.003), and the mean total skin (mean total skin difference − 0.287; *p* = 0.001).

### Length of follow-up and sensory recovery

The length of follow-up in months was significantly associated with lower monofilament values in the native, flap, and total skin of the reconstructed breasts, even before adjustment for clinically relevant variables (*p* ≤ 0.001). This applied to the innervated as well as to the non-innervated flaps (Table 5).

**Table 5:** Crude and adjusted regression coefficients of the association between length of follow-up and sensory recovery in the reconstructed breasts

	Crude coefficients	95% CI	*p* value	Adjusted coefficients^†^	95% CI	*p* value
*Innervated*						
Native skin	− 0.046	− 0.074 to − 0.019	*0.001*	− 0.055	− 0.089 to − 0.021	*0.001*
Flap skin	− 0.046	− 0.067 to − 0.024	* < 0.001*	− 0.049	− 0.072 to − 0.026	* < 0.001*
Total skin	− 0.048	− 0.071 to − 0.025	* < 0.001*	− 0.053	− 0.077 to − 0.030	* < 0.001*
*Non-innervated*						
Native skin	− 0.034	− 0.046 to − 0.22	*0.001*	− 0.034	− 0.048 to − 0.020	* < 0.001*
Flap skin	− 0.034	− 0.054 to − 0.013	*0.001*	− 0.037	− 0.060 to − 0.014	*0.002*
Total skin	− 0.040	− 0.053 to − 0.027	* < 0.001*	− 0.041	− 0.055 to − 0.026	* < 0.001*

Statistically significant p values are indicated in *italic*

^†^Adjusted for timing of reconstruction (immediate versus delayed), type of flap (DIEP versus LTP) and history of radiotherapy

After adjustment for timing of reconstruction (immediate versus delayed), type of flap (DIEP flap versus LTP flap), and history of radiotherapy, the biggest difference could be found in the native skin: the mean decrease per month was − 0.055 in innervated flaps compared to − 0.034 in non-innervated flaps. For the total skin, these differences were smaller, but in favor of the innervated flaps with a decrease of − 0.053 per month and − 0.041 in non-innervated flaps.

**Fig. 3:**
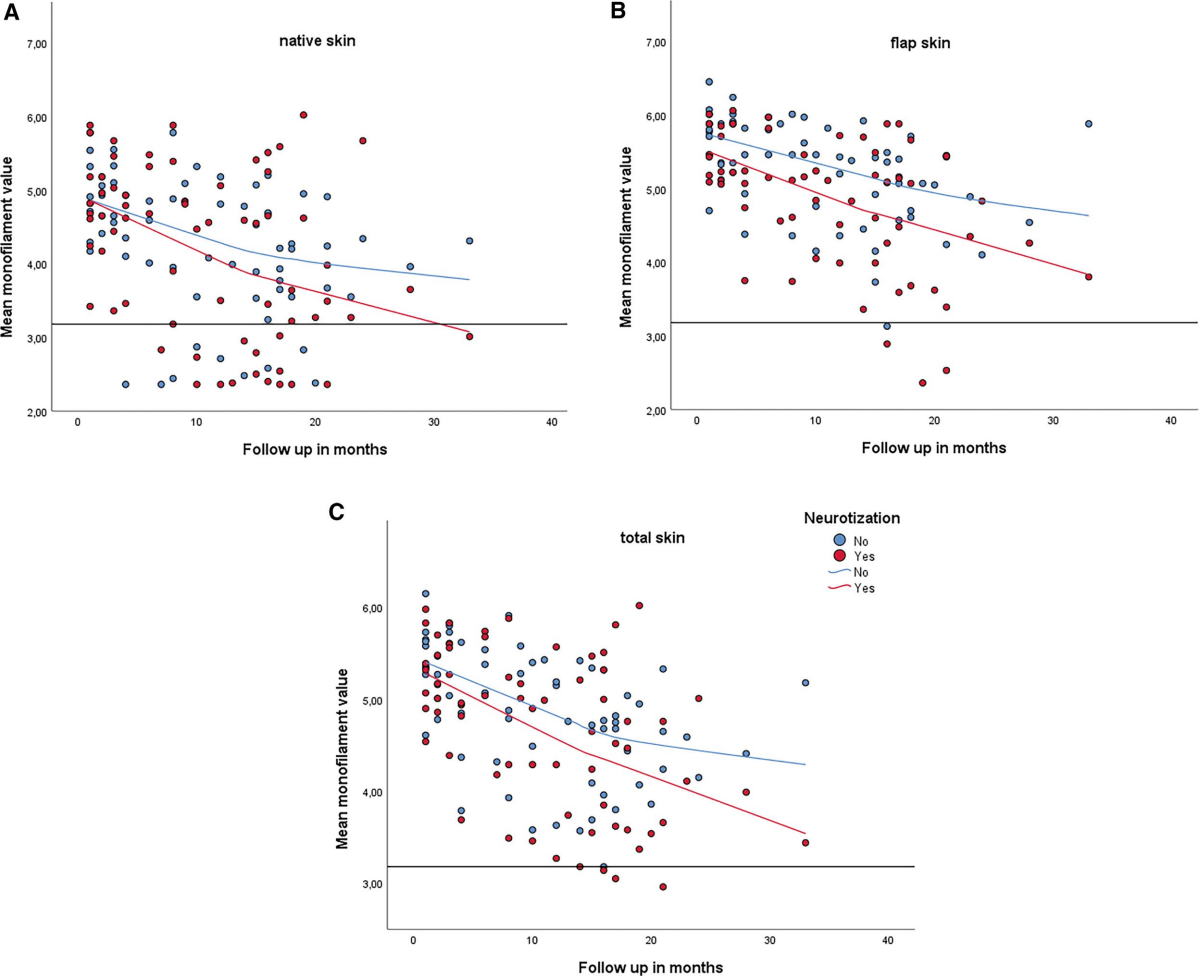
Scatterplots with all measurements of the innervated (red) and non-innervated (blue) breasts, illustrating the sensory recovery over time in the native skin (**a**), flap skin (**b**), and total skin (**c**). The black line represents the preoperative monofilament value at 3.15

The difference in sensory recovery over time between innervated and non-innervated flaps is illustrated in the scatterplots in Fig. 3.

### Clinical relevance

**Fig. 4:**
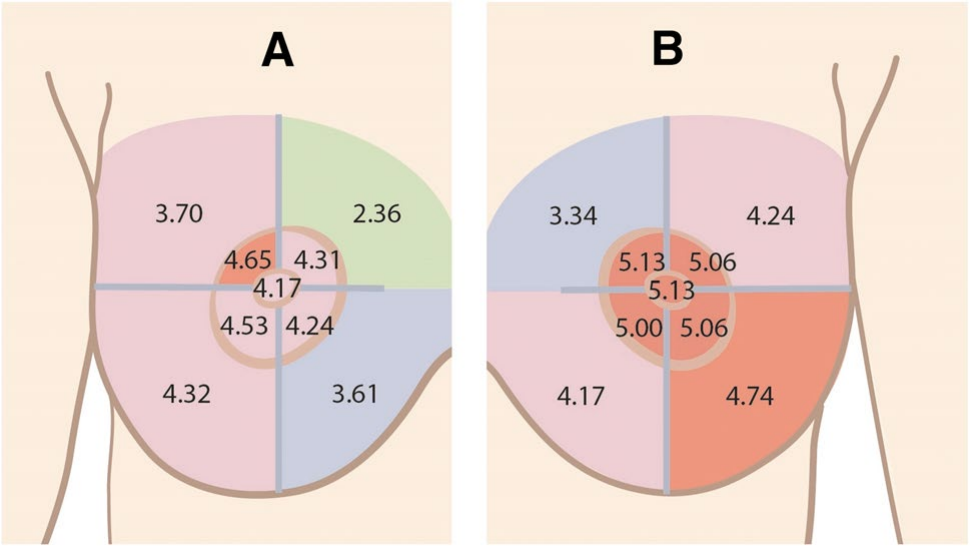
Monofilament values in breasts innervated (**a**) and non-innervated (**b**) breasts. In areas 4 and 8 (upper-outer-quadrant), the level of sensible impairment is the same in innervated and non-innervated breasts, diminished protective sensation, and loss of protective sensation, respectively. In all remaining areas, the sensory recovery was better in the innervated breasts

A schematic visualization of the levels of sensation is shown in Fig. 4. In nearly the entire breast, except area 4 and 8, sensation was more impaired in the non-innervated breasts. Moreover, in the innervated breasts (except area 8), the monofilament values reached below 4.56, indicating that the protective function of the skin in innervated breasts is preserved in the majority of the breast. In contrary, in the non-innervated breasts, the majority of the skin loses its protective function, which is indicated by the monofilament values above 4.56 (areas 3 and 5 to 9).

### Success rates sensory nerve coaptation

During the study period, a total of 122 patients underwent bilateral breast reconstruction in the Maastricht University Medical Center. Forty-nine patients underwent bilateral innervated and 52 patients underwent bilateral non-innervated breast reconstruction. Consequently, 21 patients underwent a bilateral breast reconstruction with a unilateral sensory nerve coaptation. Thus, in 70 patients (49 plus 21), it was the intention to bilaterally coaptate a sensory nerve, which equals 140 breasts. In 21 breasts (15%), a successful sensory nerve coaptation was not achieved. Almost half of the breasts in which sensory nerve coaptation was intended but not achieved were performed by one surgeon (42.9%, Table 6).

**Table 6:** Contribution of reconstructive surgeons to the unsuccessful sensory nerve coaptations

	Surgeon 1	Surgeon 2	Surgeon 3	Surgeon 4	Surgeon 5
Unsuccessful coaptations *n* = 21 (%)	9 (42.9%)	1 (4.8%)	6 (28.6%)	2 (9.5%)	3 (14.3%)

**Fig. 5:**
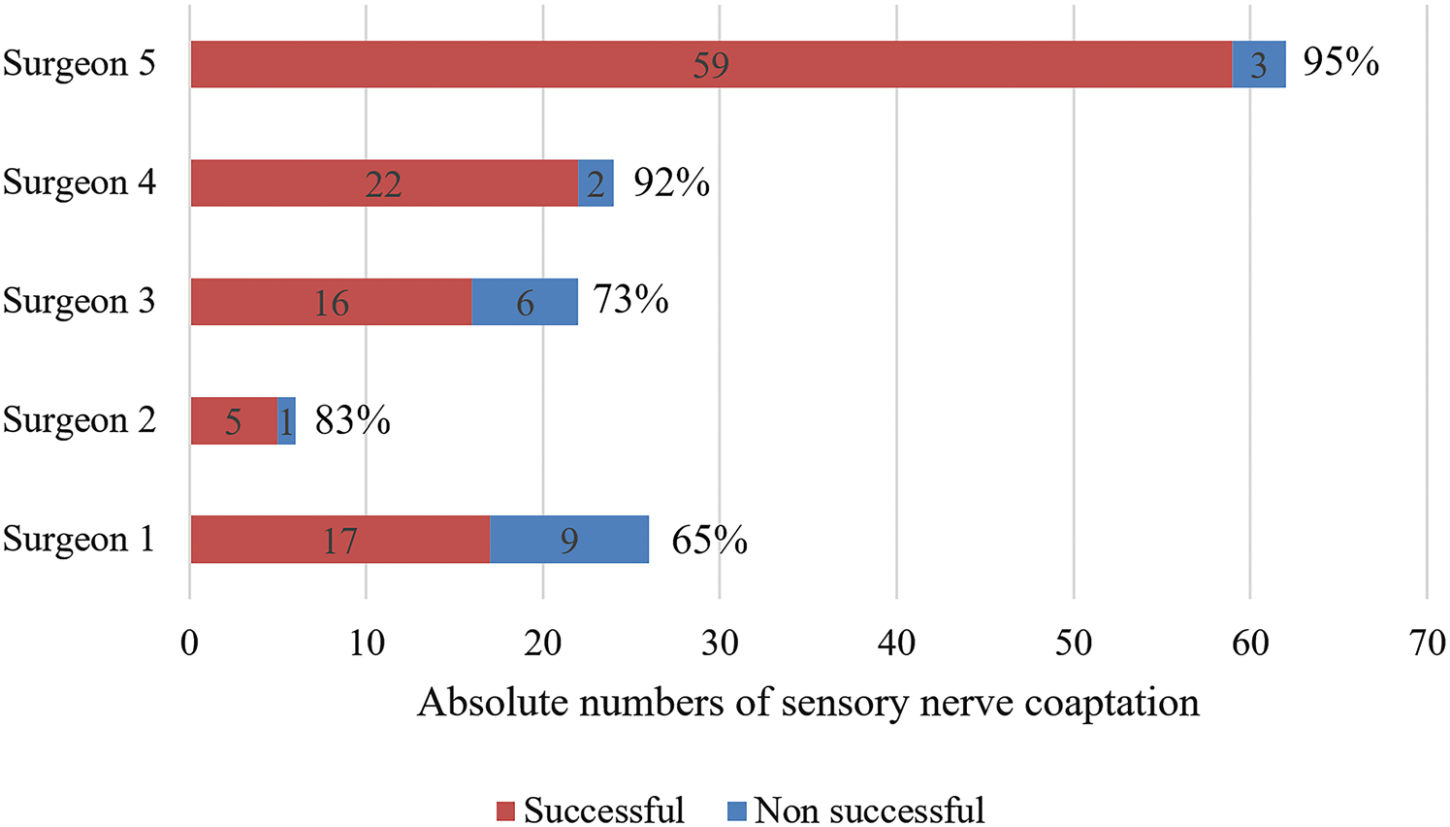
Success rates of participating reconstructive surgeons

Consequently, 85% bilateral sensory nerve coaptation was successful. The success rates between the reconstructive surgeons performing the technique of sensory nerve coaptation are shown in Fig. 5, varying from 65 to 95%. It shows that 45% of all sensory nerve coaptation attempts (62 of 140 breasts) were performed by one surgeon. Moreover, this surgeon had the highest rate of successful nerve coaptations.

## Discussion

The primary goal of this study was to evaluate the sensory recovery in reconstructed breasts in patients who underwent bilateral breast reconstruction and received one innervated and one non-innervated flap, while this was not the intention beforehand. Secondary objectives were to evaluate the association of the length of follow-up and breast sensibility and to assess the success rates of intended sensory nerve coaptations.

The current study confirms previous results that the sensory recovery in the innervated reconstructed breasts is superior to that in non-innervated breasts [6, 18], especially in the flap skin. Moreover, a longer follow-up was significantly associated with a lower monofilament value in both innervated and non-innervated breasts. Over time, the sensory recovery in the innervated breasts was significantly better in both native and flap skin. The presence of preoperative measurements is one of the strengths of this study, and so is the substantial follow-up time. The postoperative monofilament values in non-innervated breasts were significantly higher in the majority of the breast, indicating impaired sensation. In contrast, the postoperative monofilament values in innervated breasts were not significantly elevated in a total of 5 out of 9 areas compared to the preoperative values. Thus, sensation in innervated breasts has the potential to reach preoperative levels of sensation. However, it must be noted that in the current study, the preoperative monofilament values were slightly elevated compared to the monofilament value of 2.71 in the reference group found by Beugels et al. The difference might be explained by the fact that they used the contralateral, non-operated breasts in unilateral reconstructions as reference group, where in this study the preoperative measurements were also performed on breasts that had already been operated before [6]. Moreover, in two patients, implant-based breast reconstruction had already been performed previously, and this has recently been associated with severe loss of sensation [19]. Nonetheless, the results in Figs. 2 and 3 show that sensory recovery in innervated breasts is better and sensation is more likely to reach normal levels compared to sensation in non-innervated breasts in the native, flap, and total skin. In addition, the protective function of the skin is maintained in the majority of the innervated breasts, compared to non-innervated breasts. This difference was especially true for the flap skin, which lost its protective function in all areas in non-innervated breasts. These results might indicate that sensory nerve coaptation would be particularly beneficial in delayed breast reconstructions.

As mentioned before, donor nerve selection is mostly dependent on its distance to the perforator. Flap vascularity is the first priority and after that the orientation for the shape of the breast. A location close to the perforator provides a long donor nerve of 10–12 cm. This length is required for a tensionless direct end-to-end coaptation, which has been demonstrated to yield the best results in peripheral nerve repair compared to the use of nerve grafts or conduits [20, 21]. Since the dominant perforator in the abdomen is generally located within 3 cm of the umbilicus, it is assumed that the 10th or the 11th intercostal nerve is mainly used as the donor nerve. The cutaneous distribution of these nerves has been thoroughly described. They provide segmental innervation to the anterior abdominal wall and are known as the dermatomes [11]. According to this distribution, it could be reasoned that the flap skin (measurement points 5 to 9 in immediate and 2, 3, and 5 to 9 in delayed reconstructions) would not benefit from the nerve coaptation, as the 10th intercostal nerve only provides the most cranial border of the flap. This is, however, contradictory to the findings that sensory recovery is better in innervated flaps in both native and flap skin [6]. A possible explanation was given by Davies et al. and Yap et al., who found a neural intercostal plexus between the transverse and internal oblique muscles, consisting of fibers of two to three “segmental” nerves [22, 23]. This phenomenon is also applicable in LTP flaps. The LFCN is most often responsible for the cutaneous innervation of the entire lateral thigh [24]. However, both the LCFN and ACFN branch off from the femoral nerve that arises from the lumbar plexus (L2-L4). Lee et al. demonstrated that dermatomal areas are not autonomous zones of cutaneous sensory innervation since adjacent dermatomes overlap to a large and variable extent. The lateral thigh is one of the largest regions that show major variability and overlap [25]. The overlap of the dermatomes is clinically confirmed by the fact that our patients subjectively experience sensation in the upper-inner-quadrant of the breast when the flap skin is stimulated. They indicate a sensation at the level of the 2nd or 3rd intercostal space, where the sensory nerve coaptation has been performed. This applies to immediate reconstructions as well, with small skin islands in the middle of the flap. This indicates that the nerve coaptation was effective. The area pointed out by the patients when the flap skin was stimulated corresponds to the areas innervated by the ACBs of the 2nd and 3rd ICN [26]. We hypothesize that the recipient nerve, instead of the donor nerve, is responsible for where sensation is experienced by the patient.

One of the limitations of the study is the partially retrospective design, possibly leading to selection bias. However, in all included patients, bilateral sensory nerve coaptation was intended and prospective selection of eligible patients was not possible due to ethical aspects. It needs to be taken into account that there were confounding factors leading to the unsuccessful nerve coaptations, e.g., excessive scar tissue or post-radiation effects. These confounding factors will inevitably have affected the sensory recovery in the breast as well. Other aspects that might have led to unsuccessful nerve coaptations would be unfavorable flap orientation, a short vascular pedicle or not enough length of the recipient or donor nerves. Based on the results, it is plausible that sensible nerve coaptation requires adequate microsurgical training and that a learning curve exists for successful nerve coaptations.

## Conclusion

To the best of our knowledge, this is the first study to assess the sensory recovery in autologous reconstructed breasts in innervated and non-innervated flaps within the same population. The study demonstrates that the protective sensation of the skin in non-innervated flaps is lost in the majority of the breast, compared to innervated flaps, where the protective sensation is maintained in the whole breast effectively. Moreover, the effect of a learning curve for successful sensory nerve coaptations is demonstrated. To evaluate these aspects, larger study populations need to be assessed. Still, the core strength of this study lies in the characters of the study sample: all women contributed data to each study arm.

## Data Availability

Original, raw data are available as supporting information.
